# Physical therapists’ experiences and perceptions of antepartum and postpartum care

**DOI:** 10.1186/s12884-022-04484-7

**Published:** 2022-03-03

**Authors:** Kuan-Yin Lin, Yi-Ju Tsai, Jeng-Feng Yang, Meng-Hsing Wu

**Affiliations:** 1 Department of Physical Therapy, College of Medicine, National Cheng Kung University, No.1, University Road, Tainan, 701 Taiwan; 2 Institute of Allied Health Sciences, College of Medicine, National Cheng Kung University, Tainan, Taiwan; 3 Physical Therapy Center, National Cheng Kung University Hospital, Tainan, Taiwan; 4 Department of Obstetrics and Gynecology, College of Medicine, National Cheng Kung University, Tainan, Taiwan; 5 Department of Obstetrics and Gynecology, National Cheng Kung University Hospital, College of Medicine, National Cheng Kung University, Tainan, Taiwan

**Keywords:** Physical therapy, Pregnancy, Postpartum

## Abstract

**Background:**

Little is known about the physical therapists’ practice and perceptions toward management of pregnancy/postpartum-related disorders in Taiwan. The aim of this study was to document current practice of physical therapy in antepartum and postpartum care in Taiwan.

**Methods:**

An exploratory, cross-sectional study was conducted in registered physical therapists living in Taiwan. An anonymized online survey was designed, piloted, and advertised widely from March 2020 to March 2021. Data were analyzed using descriptive statistics, Chi square analysis and multivariate logistic regression.

**Results:**

Of the 364 respondents, 37.6% had experience of treating pregnant or postpartum women in clinical practice. The most commonly treated pregnancy/postpartum-related disorder in practice was low back pain (61.3%). All respondents reported little to moderate level of confidence in their ability to manage pregnancy/postpartum-related disorder. Being female (*p* < 0.01); working at a regional hospital (*p* = 0.01), district hospital or district teaching hospital (*p* < 0.01), clinic (*p* = 0.01) or physiotherapy clinic (*p* = 0.01); and having prior experience of treating antepartum or postpartum women (*p* < 0.01) were significantly associated with willingness to provide customer-oriented service or health education to patients/clients with pregnancy/postpartum-related disorder after controlling for other confounding variables. The most commonly reported barrier was “lack of available training” (81.3%).

**Conclusion:**

The majority of participating physical therapists indicated a positive attitude to antepartum and postpartum care but were not confident in management of various pregnancy/postpartum-related disorder in practices. The findings of this study highlight the educational needs related to antepartum and postpartum care in clinical practice for physical therapists in Taiwan.

## Background

In Taiwan there are approximately 210,000 births including live and still births each year [1]. During pregnancy and following childbirth, many women experience dramatic physical, hormonal, physiological and psychological changes [2–5] and often report problems with pelvic floor function (bladder, bowel and sexual dysfunction) [6, 7], breast (e.g. tenderness, blocked duct, engorgement and mastitis) [8, 9], musculoskeletal system (e.g. joint laxity, pelvic girdle pain, low back pain, diastasis recti, carpal tunnel syndrome) [10–13], cardiovascular system (e.g. anemia) [14], and mental health (e.g. anxiety, depression, insomnia) [15, 16]. These highly distressing symptoms can severely impact on the women’s well-being, daily activities, health-related quality of life and return to employment [17–19]. Despite the significant burden of these symptoms, women often do not seek any help for their problems from health professionals [20]. A recent study by Rouhi et al. reveals that health professionals often do not facilitate discussion of postpartum morbidities and women may struggle to access professional health care as a first line of treatment [20].

As part of a multidisciplinary team in gynecologic and obstetric care, physical therapists play an important role in management of the physical and psychological symptoms during pregnancy and postpartum period by providing advises on physical activity, teaching appropriate exercises including pelvic floor muscle training, treating musculoskeletal problems and giving specialized treatment (e.g. therapeutic ultrasound and massage) [21]. Physical activity or exercise has been recommended by international guidelines [22–24] and previous studies [25–28] as safe and effective in reducing the risk of gestational diabetes, severity of pelvic pain, and diastasis of the rectus abdominis muscle; and improving cardiorespiratory endurance, body composition, sleep, depressive symptoms, muscular strength, endurance and flexibility in women without contraindications before, during, and after pregnancy. Evidence has shown that physical therapy may be effective for pregnancy-related low back pain/pelvic girdle pain (exercises, lumbopelvic belt, craniosacral therapy, massage, spinal manipulation, chiropractic, and osteopathy) [29–31], weight gain (water, aerobic exercises) [31], breast pain and (breast massage) [32, 33], incontinence (PFMT) [34], and postpartum perineal pain and dyspareunia (therapeutic ultrasound) [35]. The Guideline of Canadian Physiotherapy Association for the Society of Obstetricians and Gynecologists of Canada recommends PFMT and core stability training with a physical therapist and physical therapist-prescribed exercises for women during and following pregnancy [36].

Despite the emerging evidence which supports the vital role of physical therapist in gynecologic and obstetric care, only few studies have provided data on the utilization of physical therapy services by obstetricians, gynecologists, and antepartum and postpartum women [37–40]. A Nigerian descriptive survey reported that 82% of obstetricians and gynecologists were aware of the role of physical therapists in antepartum care, 57% in parturition and 99% in postpartum care [39]. The majority of obstetricians and gynecologists (87%) agreed that patients with obstetric and gynecologic conditions require physical therapy services, and 94% referred patients for physical therapy [39]. In contrary, an Indian cross-sectional study indicated that only 15% of 106 pregnant women were referred to physical therapist for back care by their health care professionals [38]. Similar findings were reported in a UK study which showed that only 7% of 71 women received treatments for low back pain during pregnancy and none of those who consulted their general practitioner about low back pain were referred to physical therapy [41]. Although pregnant or postpartum women had positive attitudes to physical therapy [42] and perceived the information disseminated by physical therapists extremely helpful [43], previous studies demonstrated that women were reluctant to reveal physical and mental symptoms and seek help from health professionals during pregnancy and following childbirth due to the lack of health knowledge about antepartum/postpartum problems, influence of family and friends, acceptance of problems as part of their motherhood role, difficulty in accessing postpartum care, fear of being judged and women’s cultural context [20, 44].

According to the Statistics of Medical Care Institution’s Status and Hospital’s Utilization 2017 by Ministry of Health and Welfare (Taiwan) [45], there were a total of 5895 registered physical therapist in hospitals and clinics in Taiwan by the end of 2017. To date, it is unknown how many of these physical therapists are providing services to antepartum and postpartum women and how many referrals are received by physical therapists. A UK study has shown that some physical therapists have little knowledge of pregnancy and low confidence in treating women who are pregnant, which makes them reluctant to offer appropriate interventions as part of their treatment [46]. Research into current antepartum and postpartum physical therapy services delivered by physical therapy professionals in Taiwan is urgently needed. Knowledge of the provision of physical therapy services to women during pregnancy and after childbirth would provide clinicians and patients with clinically meaningful and valuable information. The objectives of this study were to (1) document what and how interventions are prescribed, monitored and evaluated; (2) explore physical therapists’ attitude and perception in the management of pregnancy/postpartum-related disorders in Taiwan; (3) identify barriers/facilitators perceived by physical therapists to service provision in antepartum and postpartum care; (4) evaluate whether there are differences between clinical background and work settings for willingness to prescribe interventions for pregnancy/postpartum-related disorders; (5) explore factors that influence whether or not therapists would provide interventions to patients/clients with pregnancy/postpartum-related disorder.

## Methods

### Ethics approval

Ethics approval for this study was obtained from the Institutional Review Board of National Cheng Kung University Hospital (−−/A-ER-108-221). Participation was voluntary. Informed consent was obtained from all participants and implied by their participation in the survey.

### Study population and recruitment

Physical therapists who were registered and practicing in Taiwan, understood Chinese/Mandarin and agreed to complete the questionnaire were eligible for inclusion. No exclusion criteria were applied. Purposive sampling was utilized for recruitment. An online questionnaire in the form of a google survey was e-mailed to members of the Taiwan Physical Therapy Association (*n* = 1088) [47] and Corporation Aggregate National Federation of Associations of Physical Therapists (approximately *n* = 5000) [48]. Participants were also recruited through an advertisement on social media platforms such as Instagram and Facebook and via our clinical networks. Snowball sampling was used as participants were also encouraged to forward the advertisement to other interested and relevant organizations / individuals.

### Design

This was a cross-sectional study. The STrengthening the Reporting of OBservational studies in Epidemiology statement [49] was followed. A mixture of open-ended and closed questions were included in the electronic survey, which took approximately 20-30 min to complete. The survey was mobile phone and tablet user friendly. All survey responses were anonymous.

The survey was purpose designed and the items were drawn from previous studies [50–53]. The survey was piloted on five physical therapists, who were asked to give written or verbal feedback on the survey length, design, format, questions included and possible responses to closed questions. Pilot participants received a NT$200 gift voucher to thank for their contribution to the survey design. The survey was structured into seven sections entitled (1) General Information (age, gender), (2) Profession and Education (highest level of education, how many years they have been qualified, how many years they have been working as a physical therapist, the area/s of physical therapy they predominantly work in or identify with, the city or county in which they practice and the clinical setting, did they receive any training or education about antepartum and postpartum care as part of university education [undergraduate/postgraduate], have they attend any courses relating to the antepartum and postpartum care since qualifying), (3) Current Practice (how many years of experience they have in treating antepartum and postpartum women, how many new and follow-up antepartum and postpartum patients a month do they treat on average, where are women with pregnancy/postpartum-related disorders referred from, what are the most common type of pregnancy/postpartum-related disorders they see in an average month, what are the assessments utilized, what is the treatment content for the disorder and how many treatment sessions they provide on average, (4) Attitudes (how effective are the treatments offered on a scale ranging from 1 [least effective] to 10 [most effective], and self-efficacy [physiotherapists self-efficacy questionnaire, a five-point scale with 1 indicating ‘very little confidence’ and 5 indicating ‘a lot of confidence’]) [54], (5) Barriers and Facilitators (a list of possible barriers and facilitators for implementation of clinical guidelines for pregnancy/postpartum-related disorders in daily practice, the level of importance to participants regarding those barriers and facilitators with a scale ranging from 1 [least important) to 10 [most important]], (6) Knowledge (awareness, familiarity, practical and technical skills) and (7) Interest in Future Research.

### Statistical analysis

All statistical analyses were performed using Statistical Package for Social Sciences, version 20.0 for Windows. Descriptive statistics were used to analyze the data from the questionnaires and reported as frequencies, percentage, and means (SD) as appropriate. Data were categorized into groups (i.e. participants who would or would not be willing to provide interventions to women with pregnancy/postpartum-related disorders) and compared using independent t test and chi-square analysis where appropriate. Multivariate logistic regression analysis was used to examine variables identified by univariate analysis (*p*-value < 0.25) [55] as associated with willingness to provide customer-oriented service or health education to patients/clients with pregnancy/postpartum-related disorder. All analyses were tested with a significance level of *p* < 0.05.

### Sample size calculation

Given the exploratory nature of this study, the pre-specified sample size calculation was based on an estimated population of 5895 practicing physical therapists [45] in Taiwan, with assumption of confidence interval 95% and margin of error 5%. The estimated sample size was 364. The survey was closed when the target number 364 was achieved.

## Results

### Survey responses

Of all respondents (*n* = 368), four did not meet the inclusion criterion (i.e. not practicing in Taiwan); hence, the total number of respondents included in the analysis was 364.

### Participant characteristics

Of 364 included participants, the majority were female (65.7%), with a mean age of 31.3 ± 8 years (Table 1). Fifty-six percent of participants had worked in their current profession for more than 5 years, with a range from less than 5 years to more than 35 years. The orthopedic physical therapy (56%) was the most often reported specialty among the respondents. Participants worked in a broad variety of settings; clinic (36.3%) was the most common. Many participants did not have access to training or education specific to antepartum and postpartum care during undergraduate or postgraduate study (69%) and after qualification (73.4%). Approximately 38% of participants had prior experience of treating antepartum or postpartum women.

**Table 1:** Characteristics of participants

Variables	n (%)
**Socio-demographics**	
Age, mean (SD)	31.3 (8.0)
Sex	
Male	125 (34.3)
Female	239 (65.7)
Marital status	
Married	121 (33.2)
Single	239 (65.7)
Living with partner	3 (0.8)
Divorced	1 (0.3)
Number of Births	
0	276 (75.8)
1	42 (11.5)
1	42 (11.5)
2	42 (11.5)
3	4 (1.1)
Highest level of education	
Associate Degree	38 (10.4)
Bachelor Degree	244 (67.0)
Master degree	69 (19.0)
PhD degree	13 (3.6)
Year of working experience (PT)	
< 5	160 (44.0)
5-10	104 (28.6)
11-15	53 (14.6)
16-20	21 (5.8)
21-25	14 (3.8)
26-30	6 (1.6)
31-35	5 (1.4)
> 35	1 (0.3)
Predominant physical therapy practice specialties	
Orthopedic	204 (56.0)
Neurology	36 (9.9)
Pediatric	26 (7.1)
Cardiovascular & Pulmonary	7 (1.9)
No specialty	81 (22.3)
Women’s Health	5 (1.4)
Sports	4 (1.1)
Oncology	1 (0.3)
Clinical setting	
Medical Center	28 (7.7)
Regional Hospital	35 (9.6)
District Hospital	55 (15.1)
District Teaching Hospital	17 (4.7)
Clinic	132 (36.3)
Physiotherapy Clinic	55 (15.1)
Educational setting (PT or relevant field)	10 (2.7)
Other	32 (8.8)
Work location^a^	
North	189 (51.8)
Center	44 (12.1)
South	122 (33.6)
East	6 (1.6)
Offshore islands	3 (0.8)
Had accessed training or education specific to antepartum and postpartum care during undergraduate or postgraduate study	
Yes	113 (31.0)
No	251 (69.0)
Had accessed courses or training specific to the antepartum and postpartum care after qualification	
Yes	97 (26.6)
No	267 (73.4)
Prior experience of treating antepartum or postpartum women	
Yes	137 (37.6)
No	227 (62.4)

*PT* physical therapy, *SD* standard deviation, *n* number, % percentage

^a^ North: Taipei, Keelung, Taoyuan, Hsinchu, Yilan; Center: Miaoli, Taichung, Changhua, Nantou, Yunlin; South: Chiayi, Tainan, Kaohsiung, Pingtung; East: Hualien and Taitung

### Current physical therapy practice for antepartum and postpartum women

Of 137 participants who had experience of working with patients during and after pregnancy, the majority had less than five new or follow-up antepartum and postpartum patients treated per month. The most common referral sources were physiatrists (54.7%), orthopedists (40.9%) and obstetrician and gynecologists (26.3%). Low back pain (61.3%), breast problems (including breast engorgement, blocked duct and breast pain) (12.4%), and pelvic pain (11.7%) were the most common referral diagnoses. Many of the respondents used various assessment tools. Respondents identified health education as the most commonly prescribed treatment for this population, followed by the therapeutic exercise and manual therapy. The mean frequency of service delivery per diagnosis was 4.1 ± 3.3, and the mean score of the perceived treatment effect was 7 out of 10 (Table 2).

**Table 2:** Current physical therapy practice for antepartum and postpartum women (*N* = 137)

Variables	n (%)
New antepartum and postpartum patients treated per month (on average)	
< 5	124 (90.5)
5-9	6 (4.4)
10-19	4 (2.9)
20-29	1 (0.7)
> 49	2 (1.5)
Follow-up antepartum and postpartum patients per month (on average)	
< 5	130 (94.9)
5-9	2 (1.5)
10-19	2 (1.5)
20-29	1 (0.7)
> 49	1 (0.7)
Women with pregnancy/postpartum-related disorders were referred from	
Division of Orthopedics	56 (40.9)
Division of Neurology	4 (2.9)
Division of Neurosurgery	3 (2.2)
Division of Plastic Surgery	1 (0.7)
Division of General Medicine	1 (0.7)
Division of Geriatrics and Gerontology	1 (0.7)
Division of Urology	2 (1.5)
Division of Family Medicine	6 (4.4)
Division of Rehabilitation Medicine	75 (54.7)
Division of Obstetrics & Gynecology	36 (26.3)
Other	20 (14.6)
Most common type of pregnancy/postpartum-related disorders seen in an average month	
Bladder dysfunction (including urinary incontinence)	11 (8.0)
Breast engorgement	7 (5.1)
Blocked duct	9 (6.6)
Breast pain	1 (0.7)
laxity	2 (1.5)
Low back pain	84 (61.3)
Pelvic pain	16 (11.7)
Diastasis recti	1 (0.7)
Carpal tunnel syndrome	4 (2.9)
Other	2 (1.5)
Assessment^a^	
History taking	130 (94.9)
Observation	116 (84.7)
Palpation	116 (84.7)
Examination of swelling	33 (24.1)
Pain assessment	93 (67.9)
Mobility test, range of motion	71 (51.8)
Muscle strength / endurance test	57 (41.6)
Joint play movement test	41 (29.9)
Neurological test	43 (31.4)
Flexibility test	56 (40.9)
Functional activity test	74 (54)
Special test	48 (35)
Disease-specific questionnaires	10 (7.3)
Other	3 (2.2)
Physiotherapy intervention provided^a^	
Therapeutic exercise	105 (76.6)
Manual therapy	105 (76.6)
Modality	72 (52.6)
Taping	68 (49.6)
Health education	110 (80.3)
Other	2 (1.5)
Frequency of service delivery per diagnosis, mean (SD)	4.1 (3.3)
Treatment effects (0-10), mean (SD)	7.0 (1.7)

*n* number, *SD* standard deviation

^a^ Participants could select multiple responses

### Self-efficacy and perception of physical therapists in managing patients with pregnancy/postpartum-related disorder

The frequency of responses in physical therapist self-efficacy can be seen in Table 3. More than half of the respondents scored their self-efficacy at three or above for most items on the scale, except for item 1 (I feel adequately prepared to undertake a pregnancy/postpartum-related disorder caseload), item 10 (I feel that I am able to perform discharge planning for a pregnancy/postpartum-related disorder caseload) and item 13 (I feel that I am able to deal with the range of patient conditions which may be seen with a pregnancy/postpartum-related disorder caseload). Overall, participants reported a little to moderate level of confidence in their ability to manage pregnancy/postpartum-related disorder (median range: 2-3).

**Table 3:** Physiotherapist Self-Efficacy questionnaire

Item	Very little confidence →A lot of confidence				
	1, n (%)	2, n (%)	3, n (%)	4, n (%)	5, n (%)
I feel adequately prepared to undertake a pregnancy/postpartum-related disorder caseload.	22 (6.0)	164 (45.1)	122 (33.5)	47 (12.9)	9 (2.5)
I feel that I am able to verbally communicate effectively and appropriately for a pregnancy/postpartum-related disorder caseload.	12 (3.3)	85 (23.4)	146 (40.1)	102 (28.0)	19 (5.2)
I feel that I am able to communicate in writing effectively and appropriately for a pregnancy/postpartum-related disorder caseload.	16 (4.4)	101 (27.7)	147 (40.4)	89 (24.5)	11 (3.0)
I feel that I am able to perform subjective assessments for a pregnancy/postpartum-related disorder caseload.	17 (4.7)	111 (30.5)	145 (39.8)	73 (20.1)	17 (4.9)
I feel that I am able to perform objective assessments for a pregnancy/postpartum-related disorder caseload.	24 (6.6)	140 (38.5)	114 (31.3)	69 (19.0)	17 (4.7)
I feel that I am able to interpret assessment findings appropriate for a pregnancy/postpartum-related disorder caseload.	19 (5.2)	142 (39.0)	123 (33.8)	63 (17.3)	17 (4.7)
I feel that I am able to identify and prioritize patients’ problems for a pregnancy/postpartum-related disorder caseload.	24 (6.6)	143 (39.3)	118 (32.4)	65 (17.9)	14 (3.8)
I feel that I am able to select appropriate short- and long-term goals for a pregnancy/postpartum-related disorder caseload.	18 (4.9)	159 (43.7)	104 (28.6)	65 (17.9)	18 (4.9)
I feel that I am able to appropriately perform treatments for a pregnancy/postpartum-related disorder caseload.	19 (5.2)	139 (38.2)	122 (33.5)	70 (19.2)	14 (3.8)
I feel that I am able to perform discharge planning for a pregnancy/postpartum-related disorder caseload.	23 (6.3)	166 (45.6)	105 (28.8)	62 (17.0)	8 (2.2)
I feel that I am able to evaluate my treatments for a pregnancy/postpartum-related disorder caseload.	17 (4.7)	152 (41.8)	113 (31.0)	64 (17.6)	18 (4.9)
I feel that I am able to progress interventions appropriately for a pregnancy/postpartum-related disorder caseload.	16 (4.4)	144 (39.6)	119 (32.7)	70 (19.2)	15 (4.1)
I feel that I am able to deal with the range of patient conditions which may be seen with a pregnancy/postpartum-related disorder caseload.	34 (9.3)	192 (52.7)	92 (25.3)	37 (10.2)	9 (2.5)

*n* number, % percentage

**Fig. 1:**
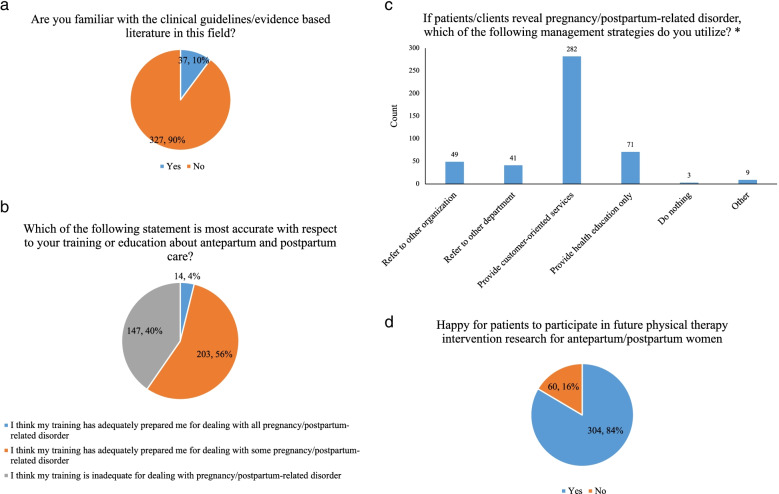
Perception of physical therapists * Participants could select multiple responses

Only 10% of participants reported that they were familiar with the clinical guidelines or evidence-based literature in antepartum and postpartum care (Fig. 1). Many participants (55.8%) considered their training had adequately prepared them for dealing with some pregnancy/postpartum-related disorder, and if patients/clients revealed pregnancy/postpartum-related disorder, the majority (77.5%) would provide customized services (Fig. 1). The majority (83.5%) of participants responded that they would be happy for their patients to participate in future physical therapy intervention research for antepartum/postpartum women.

### Barriers and facilitators

**Fig. 2:**
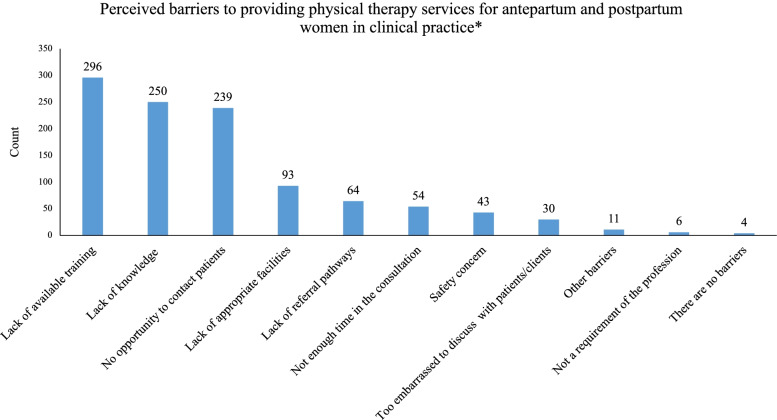
Perceived barriers to providing physical therapy services for antepartum and postpartum women in clinical practice* * Participants could select multiple responses

**Fig. 3:**
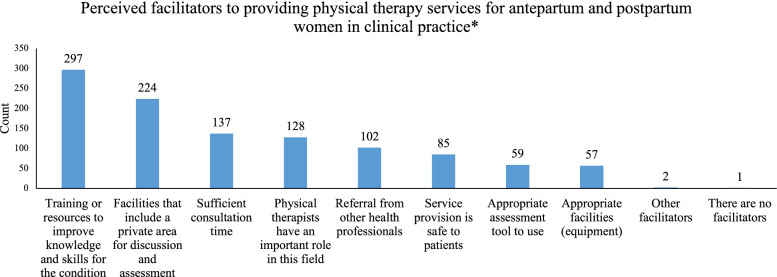
Perceived facilitators to providing physical therapy services for antepartum and postpartum women in clinical practice* * Participants could select multiple responses

The top three barriers perceived by physical therapists to service provision in antepartum and postpartum care were (a) lack of available training for physical therapists (81.3%), (b) lack of knowledge to manage pregnancy/postpartum-related disorder (68.7%), and (c) lack of opportunity to contact patients (65.7%) (Fig. 2). The top three facilitators to what would assist physical therapists most in providing services for antepartum and postpartum women were (a) training or resources to improve knowledge and skills for the condition (81.6%), (b) facilities that include a private area for discussion and assessment (61.5%), and (c) sufficient consultation time (37.6%) (Fig. 3).

### Willingness to provide interventions for women with pregnancy/postpartum-related disorders

Chi-square analysis showed that female therapists (*p* = 0.008), therapists who had accessed courses or training specific to the antepartum and postpartum care after qualification (*p* = 0.037) and therapists who had prior experience of treating antepartum or postpartum women (*p* = 0.002) were more likely to provide physical therapy or health education for pregnancy/postpartum-related disorders (Table 4). Multivariate logistic regression analysis showed that being female (*p* < 0.01); working at a regional hospital (*p* = 0.02), district hospital or district teaching hospital (*p* < 0.01), clinic (*p* = 0.01) or physiotherapy clinic (*p* = 0.02); and having prior experience of treating antepartum or postpartum women (*p* < 0.01) were significantly associated with willingness to provide customer-oriented services or health education to patients/clients with pregnancy/postpartum-related disorder after controlling for gender, age, clinical setting, had accessed training or education specific to antepartum and postpartum care during undergraduate or postgraduate study, had accessed courses or training specific to the antepartum and postpartum care after qualification, and prior experience of treating antepartum or postpartum women. The *p* value of the Hosmer and Lemeshow Goodness-of-fit test was 0.35 for the model, indicating good fitness of the models (Table 5).

**Table 4:** Characteristics of participants who would or would not be willing to provide interventions

Variables	If patients/clients reveal pregnancy/postpartum-related disorder, would you provide physical therapy or education?		Chi-square analysis *p*-value
	Yes, n (%)	No, n (%)	
Age^a^, mean (SD)	31.1 (7.8)	32.7 (9.0)	0.210
Sex			0.008
Male	102 (31.9)	23 (52.3)	
Female	218 (68.1)	21 (47.7)	
Marital status			0.250
Married	103 (32.2)	18 (40.9)	
Single	217 (67.8)	26 (59.1)	
Number of Births			0.402
0	247 (77.2)	29 (65.9)	
1	35 (10.9)	7 (15.9)	
2	36 (10.9)	7 (15.9)	
3	3 (0.9)	1 (2.3)	
Year of working experience (PT)			0.444
< 5	143 (44.7)	17 (38.6)	
5-15	139 (43.4)	18 (40.9)	
16-25	28 (8.8)	7 (15.9)	
> 25	10 (3.1)	2 (4.5)	
Clinical setting			0.152
Medical Center	23 (7.2)	5 (11.4)	
Regional Hospital	33 (10.3)	2 (4.5)	
District Hospital or District Teaching Hospital	65 (20.3)	7 (15.9)	
Clinic	116 (36.3)	16 (36.4)	
Physiotherapy Clinic	51 (15.9)	4 (9.1)	
Educational setting (PT or relevant field)	8 (2.5)	2 (4.5)	
Other	24 (7.5)	8 (18.2)	
Had accessed training or education specific to antepartum and postpartum care during undergraduate or postgraduate study			0.105
Yes	104 (32.5)	9 (20.5)	
No	216 (67.5)	35 (79.5)	
Had accessed courses or training specific to the antepartum and postpartum care after qualification			0.037
Yes	91 (28.4)	6 (13.6)	
No	229 (71.6)	38 (86.4)	
Prior experience of treating antepartum or postpartum women			0.002
Yes	130 (40.6)	7 (15.9)	
No	190 (59.4)	37 (84.1)	

*SD* standard deviation, *n* number, *SD* standard deviation, *PT* physical therapy

^a^Independent t-test

**Table 5:** Factors influencing physical therapists’ willingness to provide customer-oriented service to patients/clients with pregnancy/postpartum-related disorder

Variables	Crude OR (95% CI)	*p*-value	aOR (95% CI)^a^	*p-*value
Gender				
Male	1.0		1.0	
Female	2.34 (1.24-4.42)	< 0.01	2.95 (1.44-6.02)	< 0.01
Age, years	0.98 (0.94-1.01)	0.21	0.98 (0.93-1.02)	0.09
Marital status				
Single	1.0			
Married	0.69 (0.36-1.31)	0.25		
Number of births				
0	1.0			
1	0.59 (0.24-1.44)	0.25		
2	0.59 (0.24-1.44)	0.25		
3	0.35 (0.04-3.50)	0.37		
Year of working experience (PT)				
< 5	1.0			
5-15	0.92 (0.46-1.85)	0.81		
16-25	0.48 (0.18-1.25)	0.13		
> 25	0.59 (0.12-2.94)	0.52		
Clinical setting				
Medical Center	1.53 (0.44-5.38)	0.50	2.18 (0.54-8.84)	0.27
Regional Hospital	5.50 (1.07-28.25)	0.04	7.56 (1.38-41.58)	0.02
District Hospital or District Teaching Hospital	3.10 (1.01-9.46)	0.05	5.74 (1.71-19.20)	< 0.01
Clinic	2.42 (0.93-6.29)	0.07	3.94 (1.37-11.29)	0.01
Physiotherapy Clinic	4.25 (1.17-15.51)	0.03	5.61 (1.38-22.72)	0.02
Educational setting (PT or relevant field)	1.33 (0.23-7.63)	0.75	1.75 (0.19-15.69)	0.62
Other	1.0		1.0	
Had accessed training or education specific to antepartum and postpartum care during undergraduate or postgraduate study				
Yes	1.87 (0.87-4.04)	0.11	1.97 (0.82-4.70)	0.13
No	1.0		1.0	
Had accessed courses or training specific to the antepartum and postpartum care after qualification				
Yes	2.52 (1.03-6.16)	0.04	1.78 (0.64-4.89)	0.26
No	1.0		1.0	
Prior experience of treating antepartum or postpartum women				
Yes	3.62 (1.56-8.36)	< 0.01	4.37 (1.68-11.41)	< 0.01
No	1.0		1.0	

*aOR* adjusted odds ratio, *OR* odds ratio, *CI* confidence interval, % percent, *PT* physical therapy

^a^ Adjusted for gender, age, clinical setting, had accessed training or education specific to antepartum and postpartum care during undergraduate or postgraduate study, had accessed courses or training specific to the antepartum and postpartum care after qualification, and prior experience of treating antepartum or postpartum women

## Discussion

To the best of our knowledge, this is the first study to establish current practice of physical therapy in antepartum and postpartum care in Taiwan, to identify barriers/facilitators perceived by physical therapists to service provision in antepartum and postpartum care, and to identify factors associated with willingness to prescribe interventions for pregnancy/postpartum-related disorders. This survey suggests that only 38% of physical therapists in Taiwan had prior experience of treating antepartum or postpartum women. The treatment for women with pregnancy/postpartum-related disorders was commonly multimodal. The majority of physical therapists had a positive attitude toward contributing to antepartum and postpartum care but rated their self-efficacy as low and would like to enhance their knowledge of and skills in this topic via professional trainings or courses. The results show potential for further increasing the role of physical therapist in antepartum and postpartum care in Taiwan.

Many physical therapists in Taiwan had a lack of experience, knowledge, skill and confidence in treating pregnancy or postpartum-related complaints, which is in line with the previous study [46]. Many therapists also did not have access to training or education specific to antepartum and postpartum care. Although international guidelines and literature are available to physical therapists [23, 56–59], only few therapists are familiar them. Given the importance of evidence-based practice in helping to inform clinical decision-making [60], organizations should provide easy access to web-based resources to enhance access to research evidence databases by therapists [61]. Although physical therapists have the responsibility for their own continuing professional development, lack of time and skills in searching or appraising research literature is a common barrier reported by physical therapists [61]. Despite lack of knowledge and skill in treating women with pregnancy/postpartum-related disorders, more than half of the participants considered their training had adequately prepared them for dealing with some pregnancy/postpartum-related disorder. Nevertheless, further training is required for therapists to obtain sufficient knowledge of pregnancy/postpartum-related disorder as therapists who had access to courses or training specific to the antepartum and postpartum care after qualification were more likely to provide physical therapy for women with pregnancy/postpartum-related disorder.

Barriers to service provision might be related to individual barrier (lack of knowledge) and organizational barriers (lack of available training and lack of opportunity to contact patients). Only few women with pregnancy/postpartum-related disorders were referred to therapists and this is similar to the findings of previous studies [37, 41]. Subsequently, not many therapists had experience in treating this cohort which may have an impact on physical therapists’ competence and confidence in what they can offer to these women [62, 63]. Referral patterns (e.g. more referrals to physical therapists in the acute hospital setting than to community-based therapists) have also been shown to have an impact on physical therapists’ feelings of competency and confidence [46]; hence, the referral system and pathway may be an important factor to facilitate the antepartum/postpartum care process. Currently, direct access to physical therapy services is not available in Taiwan and physical therapy services could only be provided under the referral of physicians in seven medical specialties, including rehabilitation medicine, orthopedics, neurology, neurosurgery, plastic surgery, rheumatology, and obstetrics and gynecology (only for the diagnosis of incontinence) [64]. A multidisciplinary approach to antepartum/postpartum care should involve a physician, a nurse, and a physical therapist to enhance clinical practice guidelines and improve the health of women with pregnancy/postpartum-related disorder [65–67].

### Limitations

Several limitations are noted in this study. The purposive and snowball sampling methods are subject to selection bias, which may reduce accurate representation of the current practice in Taiwan. Another potential limitation is that the proportion of female respondents (65.7%) in this study was slightly higher compared with the gender distribution of practicing physical therapists in Taiwan (58% female) [47]. Also, discrepancies in the number of responses of physical therapists of different locations may explain the rural-urban inequities and the imbalance in the workforce distribution, hence the findings may not represent all health care systems. With only 38% of physical therapists having prior experience of treating antepartum or postpartum women, the findings lack generalizability across all physical therapists in Taiwan. In addition, due to the anonymity of respondent’s identities within an online setting, it is possible that an individual may have completed multiple surveys. Although this survey identified physical therapists’ perception, attitude and perceived barriers and facilitators to antepartum and postpartum care, further qualitative, in-depth interviews could clarify their concerns about treatments (e.g. fear of litigation and harming patients) and what they think should happen for management of pregnancy/postpartum-related disorders.

### Implications for practice

The findings from this study provide valuable evidence regarding current practice of antepartum and postpartum care provided by physical therapists in Taiwan. Continuous professional training program focused on incorporating the existing evidence and clinical experience about the safety and effectiveness of physical therapy for antepartum and postpartum women is needed for physical therapists to improve their knowledge, skill and confidence in assessing and treating this population. Furthermore, lack of opportunity to contact patients is the third barrier to service provision among physical therapists. Thus, it is important to advocate the role of physical therapy in antepartum and postpartum care in healthcare organizations and communities to help develop a referral pathway for facilitating the use of physical therapy. The results of this study may also help to refine the interventions that would be used in future trials.

## Conclusions

This study provides an overview of antepartum and postpartum service provision of physical therapists in Taiwan and identifies potential barriers for improvement. Despite relatively low confidence, inadequate training, and lack of knowledge, physical therapists’ attitude towards antepartum and postpartum care was positive, indicating a need to integrate education and trainings into the undergraduate and graduate curricula. Future studies are needed to develop strategies to implement physical therapy for this population.

## Data Availability

The datasets generated and/or analysed during the current study are not publicly available due to the need to preserve respondent confidentiality but are available from the corresponding author on reasonable request.

## Abbreviation

PFMT: Pelvic floor muscle training

## References

[1] Health Promotion Administration Ministry of Health and Welfare. 2016 Statistics of Birth Reporting System. In: Health Promotion of Administration MoHaW, editor. Taipei: Health Promotion Administration, Ministry of Health and Welfare; 2017.

[2] Haslam J, Mantle J, Haslam J, Barton S, Cardozo L (2004). Chapter 2 - physiology of pregnancy. Physiotherapy in obstetrics and Gynaecology (Second Edition).

[3] Haslam J, Mantle J, Haslam J, Barton S, Cardozo L (2004). Chapter 3 - physical and physiological changes of labour and the puerperium. Physiotherapy in obstetrics and Gynaecology (Second Edition).

[4] Webb DA, Bloch JR, Coyne JC, Chung EK, Bennett IM, Culhane JF (2008). Postpartum physical symptoms in new mothers: their relationship to functional limitations and emotional well-being. Birth..

[5] Khatun M, Clavarino AM, Callaway L, Alati R, Najman JM, Williams G (2009). Common symptoms during pregnancy to predict depression and health status 14 years post partum. Int J Gynaecol Obstet.

[6] Van Geelen H, Ostergard D, Sand P (2018). A review of the impact of pregnancy and childbirth on pelvic floor function as assessed by objective measurement techniques. Int Urogynecol J.

[7] Wallwiener S, Muller M, Doster A, Kuon RJ, Plewniok K, Feller S (2017). Sexual activity and sexual dysfunction of women in the perinatal period: a longitudinal study. Arch Gynecol Obstet.

[8] Faguy K (2015). Breast disorders in pregnant and lactating women. Radiol Technol.

[9] Schytt E, Lindmark G, Waldenstrom U (2005). Physical symptoms after childbirth: prevalence and associations with self-rated health. BJOG..

[10] Gross GA, George JW (2016). Orthopedic injury in pregnancy. Clin Obstet Gynecol.

[11] Mackenzie J, Murray E, Lusher J (2018). Women's experiences of pregnancy related pelvic girdle pain: a systematic review. Midwifery..

[12] Sperstad JB, Tennfjord MK, Hilde G, Ellstrom-Engh M, Bo K (2016). Diastasis recti abdominis during pregnancy and 12 months after childbirth: prevalence, risk factors and report of lumbopelvic pain. Br J Sports Med.

[13] Meems M, Truijens S, Spek V, Visser LH, Pop VJ (2015). Prevalence, course and determinants of carpal tunnel syndrome symptoms during pregnancy: a prospective study. BJOG..

[14] Rahman MM, Abe SK, Rahman MS, Kanda M, Narita S, Bilano V (2016). Maternal anemia and risk of adverse birth and health outcomes in low- and middle-income countries: systematic review and meta-analysis. Am J Clin Nutr.

[15] Plancoulaine S, Flori S, Bat-Pitault F, Patural H, Lin JS, Franco P (2017). Sleep trajectories among pregnant women and the impact on outcomes: a population-based cohort study. Matern Child Health J.

[16] Chen HH, Lai JC, Hwang SJ, Huang N, Chou YJ, Chien LY (2017). Understanding the relationship between cesarean birth and stress, anxiety, and depression after childbirth: a nationwide cohort study. Birth..

[17] Morin M, Vayssiere C, Claris O, Irague F, Mallah S, Molinier L (2017). Evaluation of the quality of life of pregnant women from 2005 to 2015. Eur J Obstet Gynecol Reprod Biol.

[18] Backhausen M, Damm P, Bendix J, Tabor A, Hegaard H (2018). The prevalence of sick leave: reasons and associated predictors - a survey among employed pregnant women. Sex Reprod Healthc.

[19] Lin YH, Chang SD, Hsieh WC, Chang YL, Chueh HY, Chao AS (2018). Persistent stress urinary incontinence during pregnancy and one year after delivery; its prevalence, risk factors and impact on quality of life in Taiwanese women: an observational cohort study. Taiwan J Obstet Gynecol.

[20] Rouhi M, Stirling C, Ayton J, Crisp EP (2019). Women's help-seeking behaviours within the first twelve months after childbirth: a systematic qualitative meta-aggregation review. Midwifery..

[21] Mantle J, Mantle J, Haslam J, Barton S, Cardozo L (2004). Introduction. Physiotherapy in obstetrics and Gynaecology (second edition).

[22] Artal R (2016). Exercise in pregnancy: guidelines. Clin Obstet Gynecol.

[23] Davies GAL, Wolfe LA, Mottola MF, MacKinnon C (2018). No. 129-exercise in pregnancy and the postpartum period. J Obstet Gynaecol Can.

[24] Wolfe LA, Davies GA, School of P, Health education DoO, Gynaecology, physiology QsUKOC (2003). Canadian guidelines for exercise in pregnancy. Clin Obstet Gynecol.

[25] Benjamin DR, van de Water AT, Peiris CL (2014). Effects of exercise on diastasis of the rectus abdominis muscle in the antenatal and postnatal periods: a systematic review. Physiotherapy..

[26] Davenport MH, Marchand AA, Mottola MF, Poitras VJ, Gray CE, Jaramillo Garcia A (2019). Exercise for the prevention and treatment of low back, pelvic girdle and lumbopelvic pain during pregnancy: a systematic review and meta-analysis. Br J Sports Med.

[27] Baker JH, Rothenberger SD, Kline CE, Okun ML (2018). Exercise during early pregnancy is associated with greater sleep continuity. Behav Sleep Med.

[28] Poyatos-Leon R, Garcia-Hermoso A, Sanabria-Martinez G, Alvarez-Bueno C, Cavero-Redondo I, Martinez-Vizcaino V (2017). Effects of exercise-based interventions on postpartum depression: a meta-analysis of randomized controlled trials. Birth..

[29] Hall H, Cramer H, Sundberg T, Ward L, Adams J, Moore C (2016). The effectiveness of complementary manual therapies for pregnancy-related back and pelvic pain: a systematic review with meta-analysis. Medicine (Baltimore).

[30] Franke H, Franke JD, Belz S, Fryer G (2017). Osteopathic manipulative treatment for low back and pelvic girdle pain during and after pregnancy: a systematic review and meta-analysis. J Bodyw Mov Ther.

[31] Van Kampen M, Devoogdt N, De Groef A, Gielen A, Geraerts I (2015). The efficacy of physiotherapy for the prevention and treatment of prenatal symptoms: a systematic review. Int Urogynecol J.

[32] Ahn S, Kim J, Cho J (2011). Effects of breast massage on breast pain, breast-milk sodium, and newborn suckling in early postpartum mothers. J Korean Acad Nurs.

[33] Heberle AB, de Moura MA, de Souza MA, Nohama P (2014). Assessment of techniques of massage and pumping in the treatment of breast engorgement by thermography. Rev Lat Am Enfermagem.

[34] Woodley SJ, Boyle R, Cody JD, Morkved S, Hay-Smith EJC (2017). Pelvic floor muscle training for prevention and treatment of urinary and faecal incontinence in antenatal and postnatal women. Cochrane Database Syst Rev.

[35] Hay-Smith EJ. Therapeutic ultrasound for postpartum perineal pain and dyspareunia. Cochrane Database Syst Rev. 2000;1998(2):CD000495. 10.1002/14651858.CD000495. 10.1002/14651858.CD000495 PMC7043270 10796210

[36] Britnell SJ, Cole JV, Isherwood L, Sran MM, Britnell N, Burgi S (2005). Postural health in women: the role of physiotherapy. J Obstet Gynaecol Can.

[37] Bolarinde SO, Olagunju TJ, Olley JP. Knowledge and Perception of Female Health Care Professionals on the Importance of Physiotherapy in Ante-Natal and Post-Natal Care. J Family Med Community Health. 2018;5(4):1157.

[38] Nayak R, Paes L, Gupta C, Kumar VK, Narayan A, Thunga S (2015). Knowledge, perception, and attitude of pregnant women towards the role of physical therapy in antenatal care - a cross sectional study. Online J Health Allied Scs.

[39] Odunaiya NA, Ilesanmi T, Fawole AO, Oguntibeju OO (2013). Attitude and practices of obstetricians and gynecologists towards involvement of physiotherapists in management of obstetric and gynecologic conditions. Int J Women's Health.

[40] Sarfraz M, Islami D, Hameed U, Danish S, Ahmad F, Syed (2013). Role of physical therapy in antenatal care as perceived by the clients -a cross sectional survey on pregnant females attending antenatal OPD. Pakistan J Med Dentistry.

[41] Mota MJ, Cardoso M, Carvalho A, Marques A, Sa-Couto P, Demain S (2015). Women's experiences of low back pain during pregnancy. J Back Musculoskelet Rehabil.

[42] Afroz F (2015). Pregnant women’s awareness about physiotherapy services at selected maternity hospital.

[43] Hermansen IL, O'Connell B, Gaskin CJ (2010). Are postpartum women in Denmark being given helpful information about urinary incontinence and pelvic floor exercises?. J Midwifery Womens Health.

[44] Mason L, Glenn S, Walton I, Hughes C (2001). Women's reluctance to seek help for stress incontinence during pregnancy and following childbirth. Midwifery..

[45] Statistics of Medical Care Institution's Status & Hospital Utilization 2017. Taipei: Ministry of Health and Welfare; 2018. https://www.mohw.gov.tw/lp-4113-2.html. Accessed 4 June 2021.

[46] Waterfield J, Bartlam B, Bishop A, Holden MA, Barlas P, Foster NE (2015). Physical Therapists' views and experiences of pregnancy-related low Back pain and the role of acupuncture: qualitative exploration. Phys Ther.

[47] World Physiotherapy 2021 (2021). Annual membership census 2021- TAIWAN.

[48] Corporation Aggregate National Federation of Associations of Physical Therapists: About N.F.A.P.T. 2012. http://www.pt.org.tw/english/. Accessed 4 June 2021.

[49] von Elm E, Altman DG, Egger M, Pocock SJ, Gotzsche PC, Vandenbroucke JP (2014). The Strengthening the reporting of observational studies in epidemiology (STROBE) statement: guidelines for reporting observational studies. Int J Surg.

[50] Comer CM, Redmond AC, Bird HA, Conaghan PG (2009). Assessment and management of neurogenic claudication associated with lumbar spinal stenosis in a UK primary care musculoskeletal service: a survey of current practice among physiotherapists. BMC Musculoskelet Disord.

[51] Alshehri MA, Alalawi A, Alhasan H, Stokes E (2017). Physiotherapists' behaviour, attitudes, awareness, knowledge and barriers in relation to evidence-based practice implementation in Saudi Arabia: a cross-sectional study. Int J Evid Based Healthc.

[52] Liu W-Y, Lien H-Y, Li Y-C, Chen C-N, Chen C-Y, Chen Y-C (2019). Core values of physical therapy professionalism: the viewpoints of clinical physical therapists in Taiwan. Formosan J Phys Ther.

[53] Hayman M, Short C, Reaburn P (2017). Regionally based medical practitioners may need support when prescribing exercise to pregnant women. Aust J Rural Health.

[54] Jones A, Sheppard L (2012). Developing a measurement tool for assessing physiotherapy students' self-efficacy: a pilot study. Assess Eval High Educ.

[55] Chowdhury MZI, Turin TC (2020). Variable selection strategies and its importance in clinical prediction modelling. Fam Med Community Health.

[56] Senat MV, Sentilhes L, Battut A, Benhamou D, Bydlowski S, Chantry A (2016). Postpartum practice: guidelines for clinical practice from the French College of Gynaecologists and Obstetricians (CNGOF). Eur J Obstet Gynecol Reprod Biol.

[57] Bhardwaj A, Nagandla K (2014). Musculoskeletal symptoms and orthopaedic complications in pregnancy: pathophysiology, diagnostic approaches and modern management. Postgrad Med J.

[58] Savvaki D, Taousani E, Goulis DG, Tsirou E, Voziki E, Douda H (2018). Guidelines for exercise during normal pregnancy and gestational diabetes: a review of international recommendations. Hormones (Athens).

[59] Charlesworth S, Foulds HJ, Burr JF, Bredin SS (2011). Evidence-based risk assessment and recommendations for physical activity clearance: pregnancy. Appl Physiol Nutr Metab.

[60] Majid S, Foo S, Luyt B, Zhang X, Theng YL, Chang YK (2011). Adopting evidence-based practice in clinical decision making: nurses' perceptions, knowledge, and barriers. J Med Libr Assoc.

[61] Scurlock-Evans L, Upton P, Upton D (2014). Evidence-based practice in physiotherapy: a systematic review of barriers, enablers and interventions. Physiotherapy..

[62] Bent A (1999). Allied health in Central Australia: challenges and rewards in remote area practice. Aust J Physiother.

[63] Minisini M, Sheppard LA, Jones A (2010). Self-efficacy beliefs and confidence of rural physiotherapists to undertake specialist paediatric caseloads: a paediatric example. Rural Remote Health.

[64] Liao H-F, Wang S-F, Chai H-M (2011). History, present status, and existing straits of physical therapy in Taiwan. Formosan J Physical Ther.

[65] Ickovics JR, Lewis JB, Cunningham SD, Thomas J, Magriples U (2019). Transforming prenatal care: multidisciplinary team science improves a broad range of maternal-child outcomes. Am Psychol.

[66] Brennen R, Sherburn M, Rosamilia A (2019). Development, implementation and evaluation of an advanced practice in continence and women's health physiotherapy model of care. Aust N Z J Obstet Gynaecol.

[67] Chalmers B (1999). Multicultural, multidisciplinary and psycho-social obstetrical care. J SOGC.

